# Thanks to our peer reviewers

**DOI:** 10.1002/lrh2.70133

**Published:** 2026-10-30

The publication of Issue 4 marks the completion of Volume 10 of *Learning Health Systems*. An international, trans‐disciplinary, open access publication, the journal has advanced research and scholarship on learning health systems in partnership with our reviewers. Articles from the journal had more than 240,000 full‐text views in 2025. We continue to diversify the offerings of the journal through special issues and special collections, and through the supplemental issue published each year in collaboration with AcademyHealth.

We are keenly aware that these achievements would not have happened without the dedicated efforts and insightful comments of all those individuals who accepted invitations to review submitted articles. With busy schedules and full commitments, these individuals found the time and energy to contribute their expertise to our authors to help ensure that their papers met (and often exceeded) the journal's high standards for publication.

Please accept our sincere gratitude for your outstanding efforts!

Charles P. Friedman, Editor in Chief.

Nancy Allee, Senior Associate Editor.

Linda Novak, Managing Editor.

***Learning Health Systems* Peer Reviewers**.

Note: These are the reviewers for articles published in all four issues of Volume 10. Reviewers of manuscripts that were not ultimately published are also included in this list.

Ibukun Abejirinde (Canada)

Julia Adler‐Milstein (United States)

Ali Alsarhan (United States)

Muhammad Amith (United States)

Holt Anderson (United States)

Luca Anselma (Italy)

Orhun Aydin (United States)

Pierre Barker (United States)

Philip Barrison (United States)

Timothy Beebe (United States)

Sarah Birken (United States)

Nicholas Bowersox (United States)

Mara Bria (Romania)

Jeffrey Brown (United States)

James Buehler (United States)

Eren Çamur (Turkey)

Michael Cantor (United States)

Yidan Cao (United States)

Sitong Chen (Australia)

Genna Cohen (United States)

Rebecca Yates Coley (United States)

Harold Collard (United States)

Marisa Conte (United States)

Eric Crosby (United States)

Theresa Cullen (United States)

Trevor Denton (United States)

Ajay Dharod (United States)

Neal Dickert (United States)

Brian Dixon (United States)

Nancy Dreyer (United States)

Douglas Easterling (United States)

Peter Embi (United States)

Keith Feldman (United States)

Stephan Fihn (United States)

Allen Flynn (United States)

Tom Foley (United Kingdom of Great Britain and Northern Ireland)

Patricia Franklin (United States)

Hamish Fraser (United States)

Sarah Greene (United States)

Antoine Groulx (Canada)

Miranda Hallquist (United States)

W. Ed Hammond (United States)

Samuel Hanke (United States)

Calista Harbaugh (United States)

Julia Harris (United States)

Richard Harrison (United States)

David Hartley (United States)

Kristi Holmes (United States)

Matthew Hudson (United States)

Lillian Hung (Canada)

Edward Hurvitz (United States)

Dipak Kalra (Belgium)

Amol Karmarkar (United States)

Anjum Khurshid (United States)

Rebecca Kinney (United States)

Robert Kolodner (United States)

Nik Koscielniak (United States)

Andrew Krumm (United States)

Zach Landis‐Lewis (United States)

Lisa Lederer (United States)

Allen Lichter (United States)

David McCallie (United States)

Paige McDonald (United States)

Kathleen McTigue (United States)

Kayla Mehl (United States)

Genevieve Melton‐Meaux (United States)

Michelle Mendiolaza (United States)

Virginia Minogue (United Kingdom)

Amanda Missel (United States)

Stephanie Morain (United States)

Cheryl Moyer (United States)

Alva Hendi Muhammad (Indonesia)

Mark Musen (United States)

Stuart Nicholls (Canada)

Andrew Nierenberg (United States)

Paige Nong (United States)

Soichi Ogishima (Japan)

Philip Payne (United States)

Gretchen Piatt (United States)

Miranda Pollock (United States)

Wayne Psek (United States)

Alanna Kulchak Rahm (United States)

Ivan Raikov (United States)

Sandeep Reddy (Australia)

Robert Reid (Canada)

Melissa Rethlefsen (United States)

Rachel Richesson (United States)

Joshua Rubin (United States)

Lucy Savitz (United States)

Emily Schindeler (Australia)

Titus Schleyer (United States)

Kenneth Scott (United States)

Philip Scott (United Kingdom of Great Britain and Northern Ireland)

Farid Seifi (United States)

Jessica Simon (Canada)

Carolynn Smith (Australia)

Caren Stalburg (United States)

Glen Taksler (United States)

Brent Taylor (United States)

Jessica Tenenbaum (United States)

Jack Tsai (United States)

Robert Verheij (Netherlands)

Kristen Vibbert (United States)

Alexandra Vinson (United States)

Chen Wang (United States)

Brittany Watson (United States)

Mark Weiner (United States)

Francesca Williamson (United States)

Brendon Wolff‐Piggott (South Africa)

Brianne Wood (Canada)

Yili Zhang (United States)

Merrick Zwarenstein (Canada)

